# Control of hyperuricemia in subjects with refractory gout, and induction of antibody against poly(ethylene glycol) (PEG), in a phase I trial of subcutaneous PEGylated urate oxidase

**DOI:** 10.1186/ar1861

**Published:** 2005-12-02

**Authors:** Nancy J Ganson, Susan J Kelly, Edna Scarlett, John S Sundy, Michael S Hershfield

**Affiliations:** 1 Division of Rheumatology, Box 3049, Duke University Medical Center, Durham, NC 27710, USA; 2 Department of Biochemistry, Box 3049, Duke University Medical Center, Durham, NC 27710, USA

## Abstract

PEG-modified recombinant mammalian urate oxidase (PEG-uricase) is being developed as a treatment for patients with chronic gout who are intolerant of, or refractory to, available therapy for controlling hyperuricemia. In an open-label phase I trial, single subcutaneous injections of PEG-uricase (4 to 24 mg) were administered to 13 such subjects (11 had tophaceous gout), whose plasma uric acid concentration (pUAc) was 11.3 ± 2.1 mg/dl (mean ± SD). By day seven after injection of PEG-uricase, pUAc had declined by an average of 7.9 mg/dl and had normalized in 11 subjects, whose mean pUAc decreased to 2.8 ± 2.2 mg/dl. At doses of 8, 12, and 24 mg, the mean pUAc at 21 days after injection remained no more than 6 mg/dl. In eight subjects, plasma uricase activity was still measurable at 21 days after injection (half-life 10.5 to 19.9 days). In the other five subjects, plasma uricase activity could not be detected beyond ten days after injection; this was associated with the appearance of relatively low-titer IgM and IgG antibodies against PEG-uricase. Unexpectedly, these antibodies were directed against PEG itself rather than the uricase protein. Three PEG antibody-positive subjects had injection-site reactions at 8 to 9 days after injection. Gout flares in six subjects were the only other significant adverse reactions, and PEG-uricase was otherwise well tolerated. A prolonged circulating life and the ability to normalize plasma uric acid in markedly hyperuricemic subjects suggest that PEG-uricase could be effective in depleting expanded tissue stores of uric acid in subjects with chronic or tophaceous gout. The development of anti-PEG antibodies, which may limit efficacy in some patients, is contrary to the general assumption that PEG is non-immunogenic. PEG immunogenicity deserves further investigation, because it has potential implications for other PEGylated therapeutic agents in clinical use.

## Introduction

Attacks of inflammatory arthritis in patients with gout are triggered by monosodium urate crystals, which result from the low solubility and high levels of uric acid in plasma and extracellular fluids [1,2]. Gout can usually be controlled by maintaining serum urate below the limit of solubility (about 7 mg/dl, or 0.42 mM) with drugs that block urate synthesis by inhibiting xanthine oxidase, or that promote renal urate excretion [3]. For various reasons (noncompliance, intolerance, inadequate dosage, or inefficacy), therapy fails in a subset of patients, who may develop destructive arthropathy, widespread deposition of urate in tissues (tophi), and nephropathy [4]. At this chronic stage, urate deposits built up over decades are only slowly depleted by blocking the synthesis of urate, particularly because the renal clearance of urate is often inefficient in these patients. The management of chronic gout may be further complicated by co-morbidities such as hypertension, heart disease, diabetes, and renal insufficiency, which may limit the use of anti-inflammatory agents to treat arthritis.

Urate levels are low and gout does not occur in species that express urate oxidase, which converts urate to the more soluble and easily excreted compound allantoin. Although in humans the uricase gene was inactivated by mutations during evolution, parenteral uricase is a potential means of controlling hyperuricemia and depleting urate stores [5,6]. Infusion of *Aspergillus flavus *uricase (Rasburicase; Sanofi Synthelabo) is used to prevent acute uric acid nephropathy caused by tumor lysis in patients with leukemia and lymphoma [7,8]. However, the 18 hour half-life, which necessitates daily infusion, and potential immunogenicity limit the long-term use of fungal uricase, which would be necessary for treating chronic gout.

Covalent attachment of PEG can prolong the circulating life and diminish the immunogenicity of proteins [9-11]. More than 15 years ago we used a PEGylated bacterial uricase on a compassionate basis to treat uric acid nephropathy in a patient with lymphoma who was allergic to the xanthine oxidase inhibitor allopurinol [12]. We have since pursued the development of a PEGylated recombinant mammalian uricase as an orphan drug for treating refractory gout. In a preclinical study, weekly administration of this mammalian PEG-uricase normalized urate levels and prevented uric acid nephropathy in a strain of mice in which the uricase gene had been disrupted [13]. Unmodified recombinant uricase was ineffective and highly immunogenic, whereas antibodies against uricase were not detected in mice repeatedly dosed with PEG-uricase.

Here we report results of the initial phase I trial in which mammalian PEG-uricase was administered by subcutaneous injection to human subjects with refractory gout. Single injections of PEG-uricase resulted in marked and prolonged lowering of plasma urate concentration. However, in several subjects the circulating life and efficacy of PEG-uricase was foreshortened by the induction of antibodies against PEG-uricase, which, unexpectedly, were specific for PEG rather than for the uricase protein. This finding conflicts with the general assumption that PEG is non-immunogenic, and it thus has potential implications for other PEGylated agents used to treat diverse diseases.

## Materials and methods

### Materials

The PEG-uricase used in this clinical trial consists of a recombinant mammalian uricase (primarily from pig, with a carboxy-terminal sequence from baboon), modified by covalent attachment of multiple strands of 10 kDa monomethoxyPEG (10 K mPEG) per subunit of the tetrameric enzyme [13]. Savient Pharmaceuticals, Inc. (East Brunswick, NJ, USA) manufactured PEG-uricase and provided it in vials containing 12 mg of PEG-uricase (195.5 units, assayed as described below) in 1 ml of a phosphate buffer. Savient also provided the unmodified recombinant mammalian uricase and *p*-nitrophenyl carbonate (NPC)-activated 10 K mPEG, which were used to study antibody specificity as described below. Other PEG preparations used in these latter studies were obtained from Sigma (St Louis, MO, USA).

### Study design and subjects

The pharmacokinetics, efficacy, immunogenicity, and safety of PEG-uricase were investigated in an open-label, single-injection (subcutaneous), dose-escalation phase I trial, which was conducted at Duke University Medical Center and sponsored by Savient Pharmaceuticals. This trial was approved by the Duke University Investigational Review Board. Study subjects had symptomatic gout (at least one flare in the previous six months, chronic arthropathy due to gout, or tophi), and a serum urate concentration of more than 7 mg/dl. Exclusion criteria included pregnancy, renal failure requiring dialysis, the use of immunosuppressive agents (other than prednisone at not more than 10 mg per day to control attacks of arthritis), a deficiency of glucose-6-phosphate dehydrogenase, or co-morbidities that might complicate the evaluation of safety. Allopurinol and uricosuric drugs were withheld for 2 weeks before, and for 21 days after, the administration of PEG-uricase by subcutaneous injection. Groups of four subjects were scheduled to receive 4, 8, 12, or 24 mg of PEG-uricase. The response to PEG-uricase was monitored for 21 days after drug administration. Because of hypersensitivity reactions observed in three subjects, the trial was stopped after one subject was enrolled in the 24 mg dose group. The results of this study have been described previously in preliminary form [14].

### Pharmacokinetics

PEG-uricase was measured as urate oxidase activity in plasma (pUox) by a modification of a previously described radiochemical HPLC assay [15]. In this modified assay, which was validated in accordance with recommended standards [16], [8-^14^C]uric acid is oxidized to [^14^C]allantoin during incubation with study plasma in borate buffer at 37°C. The ^14^C-labeled substrate and oxidation products are then separated by reverse-phase HPLC (an Agilent 1100 system equipped with a diode array detector and ChemStation software was used). Uric acid concentration in the column effluent was monitored at 292 nm and quantified by reference to a standard calibration curve. ^14^C label in column effluent was measured with a coupled flow-through radioactivity detector and LauraLite software (IN/US Systems, Tampa, FL, USA). The specific radioactivity (counts per second per pmol) of the [8-^14^C]uric acid substrate determined in this manner, which varies with urate concentration in the plasma sample, is then applied to the radioactivity (counts per second) in the oxidation product region of the chromatogram to calculate the amount (pmol) of ^14^C-labeled product formed. The rate of urate oxidation in milliunits per ml of plasma is then calculated (1 unit = 1 μmol of urate oxidized per minute).

### Pharmacodynamics

Efficacy was assessed by the magnitude of decrease in plasma uric acid concentration (pUAc). For this measurement, heparinized blood was immediately placed on ice and centrifuged at 2 to 4°C; the resulting plasma was then acidified by diluting 1:5 with 0.375 M perchloric acid to inactivate PEG-uricase. Uric acid in the acidified plasma was quantified by HPLC as described above for the pUox assay. To be consistent with clinical practice and to permit comparison with previous medical literature on gout, pUAc is expressed in 'mg/dl' (1 mM uric acid = 16.81 mg/dl; a pUAc of 7.0 mg/dl = 0.416 mM).

### ELISA to detect IgG antibody against PEG-uricase

Wells of a microtiter plate (Immunlon 2HB; Dynex Technologies, Chantilly, VA, USA) were coated overnight at 4°C with 50 μl of 50 μg/ml PEG-uricase in PBS, or with PBS ('blank'). After being washed with PBS, all wells were blocked with 1% BSA in PBS. Dilutions (1:20 to 1:60) of plasma samples were then added to duplicate wells; 1% BSA in PBS was added to 'blank' wells in quadruplicate. Plates were sealed and incubated for 1 hour at 37°C, then overnight at 4°C. After being washed with PBS containing 0.1% Tween 20, 100 μl of 1:1,000 or 1:10,000 diluted peroxidase or alkaline phosphatase-conjugated goat anti-human γ-chain-specific immunoglobulin (Sigma) was added to each well. After incubation for 1 hour at 22–25°C, plates were washed with PBS (for peroxidase-coupled reagents) or Tris-buffered saline (for phosphatase-coupled reagents) containing 0.1% Tween 20. Bound peroxidase or alkaline phosphatase was then detected by incubation, respectively, with *o*-phenylenediamine hydrochloride and hydrogen peroxide, or with *p*-nitrophenyl phosphate, in accordance with the directions of the supplier. Absorbance (*A*) at 405 nm (phosphatase reactions) or at 490 nm (peroxidase reactions) was monitored with a plate reader (Molecular Devices, Sunnyvale, CA, USA). Peroxidase reactions were terminated by adding 100 μl of 1 M HCl when the *A*_490 _of the sample with the highest signal reached about 0.2. Phosphatase reactions were terminated by adding 50 μl of 10% NaOH when *A*_405 _for this sample reached approximately 1.0. (A similar protocol was used to detect IgM antibodies, but using anti-human μ-chain-specific reagents.)

A 'positive' ELISA response was initially defined as an *A*_405 _or *A*_490 _more than 3 SD above the mean for day 0 pretreatment plasma samples from study subjects (subsequently, more than 3 SD above the mean for a panel of healthy control sera supplied by the Duke University Clinical Immunology Laboratory). Day 14 and day 21 plasma from the study subject with the highest ELISA response in the initial screen was used as a 'positive' reference in subsequent ELISAs. Studies to establish specificity for the uricase protein and various PEG preparations are described in the text and figure legends.

## Results

### Subject characteristics

The study population consisted of 13 subjects with symptomatic gout and hyperuricemia. Nine subjects were intolerant of allopurinol, or had progressed to a chronic stage despite ongoing treatment with allopurinol (Table 1). Tophi were present in 11 subjects. The serum uric acid for all subjects, measured just before allopurinol washout, was 10.1 ± 2.3 mg/dl (results are shown as means ± SD throughout) (range 6.9 to 14.7); levels were similar in the six subjects receiving allopurinol and in the seven who were not. After the two-week allopurinol washout, overall pUAc rose to 11.3 ± 2.1 mg/dl (range 7.5 to 14.9). The two subjects with the highest pUAc (14.8 and 14.9 mg/dl) were brothers with partial deficiency of hypoxanthine–guanine phosphoribosyltransferase [17]. The underlying basis for gout in the other subjects was unknown. Other characteristics of the study subjects are listed in Table 1.

### Plasma uricase activity and relation to plasma urate concentration

Before treatment, pUox was undetectable. After subcutaneous injection of PEG-uricase, pUox increased gradually, suggesting slow absorption into the circulation. The time to reach peak pUox (*t*_max_) varied from 2 to 10 days, averaging 7 days. Within each dose cohort there were large differences in highest and lowest pUox values at each time point. Maximum pUox (*C*_max_) values for the groups receiving doses of 4, 8, and 12 mg were in the ranges 4.9 to 7.5, 8.1 to 21.4, and 6.2 to 13.6 mU/ml, respectively; *C*_max _was 25.6 mU/ml in the single subject who received a 24 mg dose.

**Figure 1:**
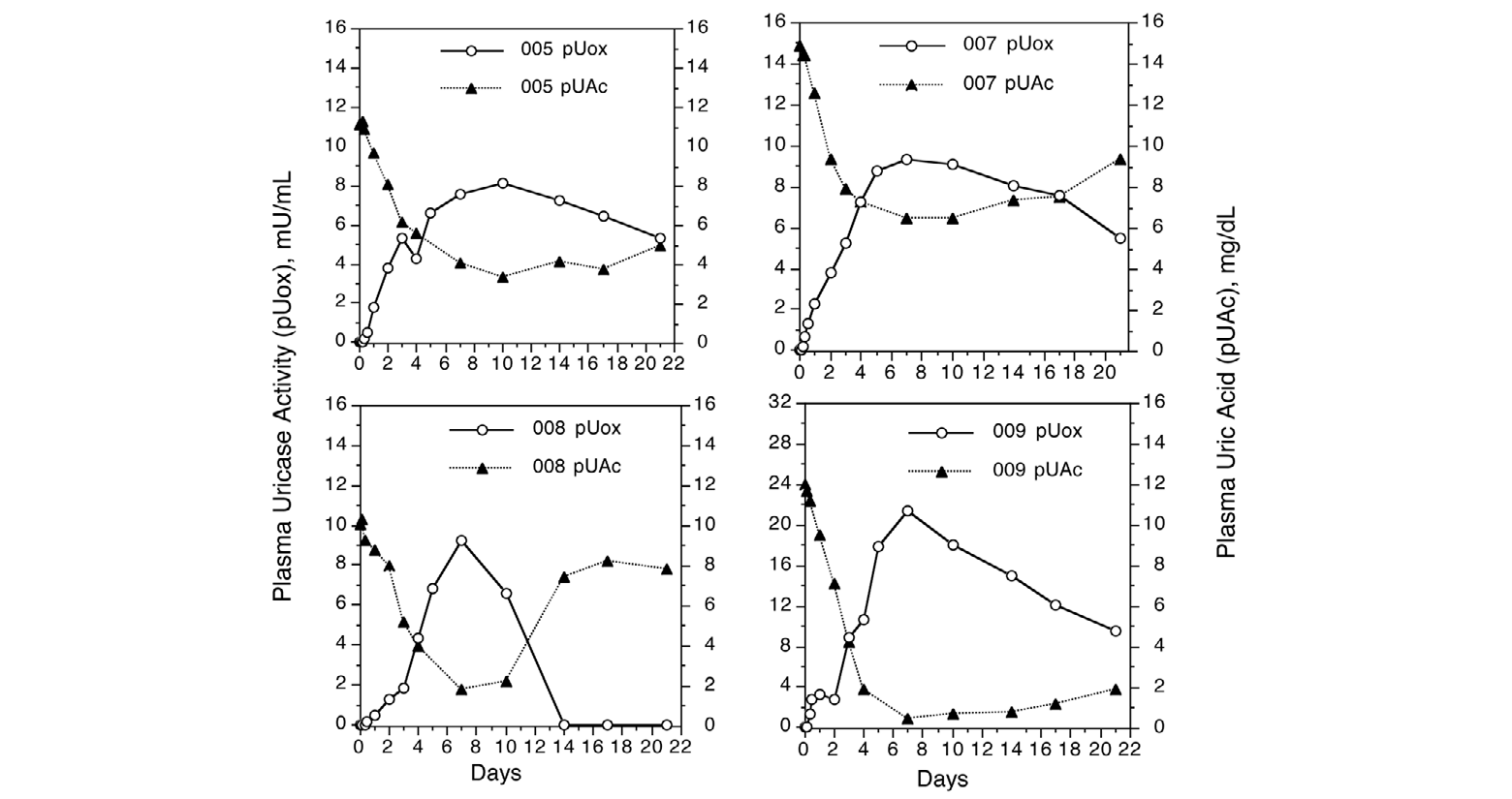
Plasma uricase activity and plasma uric acid concentration after subcutaneous injection of PEG-uricase. A single 8 mg injection of PEG-modified recombinant mammalian urate oxidase (PEG-uricase) was administered. The horizontal axis indicates days after dosing.

In spite of variable pharmacokinetics, in every subject there was a clear inverse relationship between simultaneously measured levels of pUox and pUAc (shown for the 8 mg dose cohort in Figure 1). The average time for pUAc to reach a nadir was seven days, coinciding with the *C*_max _for pUox. Overall, pUAc decreased by 7.9 ± 2.8 mg/dl from the pre-injection level; the nadir was less than 6.5 mg/dl in 11 subjects (2.8 ± 2.2 mg/dl).

**Figure 2:**
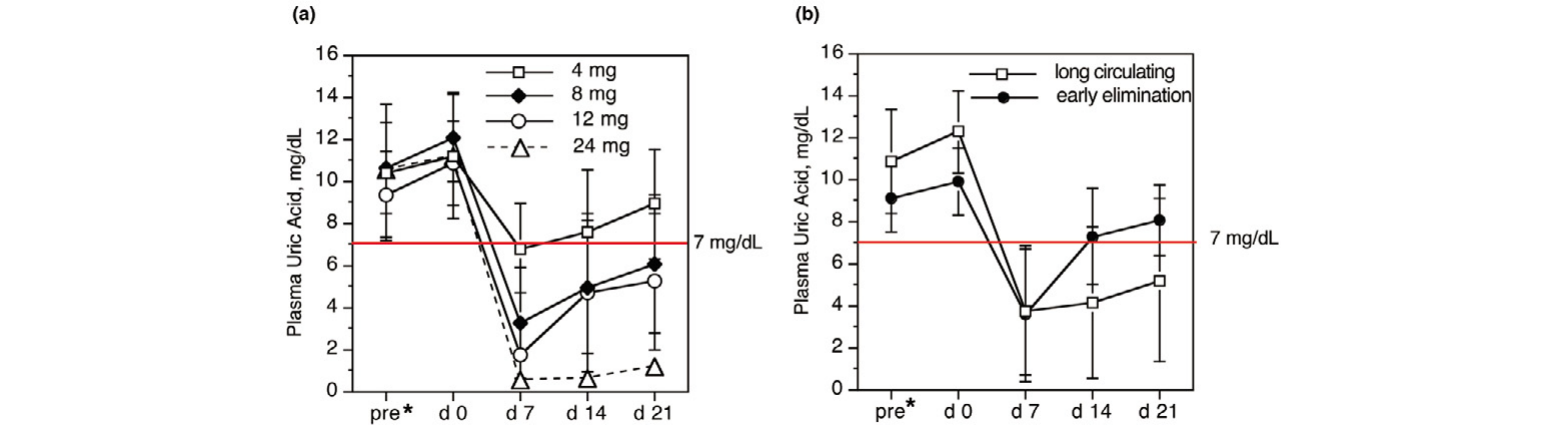
Relationship of dose of PEG-uricase and serum or plasma uric acid concentration. **(a) **Data for each dose cohort. **(b) **Data for two groups of subjects with distinct pharmacokinetic patterns: 'long-circulating' (plasma uricase activity (pUox) present three weeks after injection (*n *= 8)) and 'early elimination' (pUox undetectable beyond 10 days after injection (*n *= 5)). The horizontal axis indicates days after injection of PEG-modified recombinant mammalian urate oxidase (PEG-uricase); 'pre*', serum obtained at screening, just before the two-week allopurinol washout period; 'd 0', plasma obtained immediately before PEG-uricase injection.

The relationship of pUAc to dose of PEG-uricase and time after injection is shown in Figure 2a. In all dose cohorts the mean pUAc on day 7 was less than 7 mg/dl; values were 3.2 ± 2.6 and 1.8 ± 1.5 mg/dl for the 8 and 12 mg cohorts, respectively. At 21 days after injection the mean pUAc in the 8 and 12 mg dose cohorts was 6.0 ± 3.3 and 5.2 ± 3.2 mg/dl, respectively, and was 1.2 mg/dl in the subject treated with 24 mg of PEG-uricase.

### Two pharmacokinetic patterns of PEG-uricase elimination

**Figure 3:**
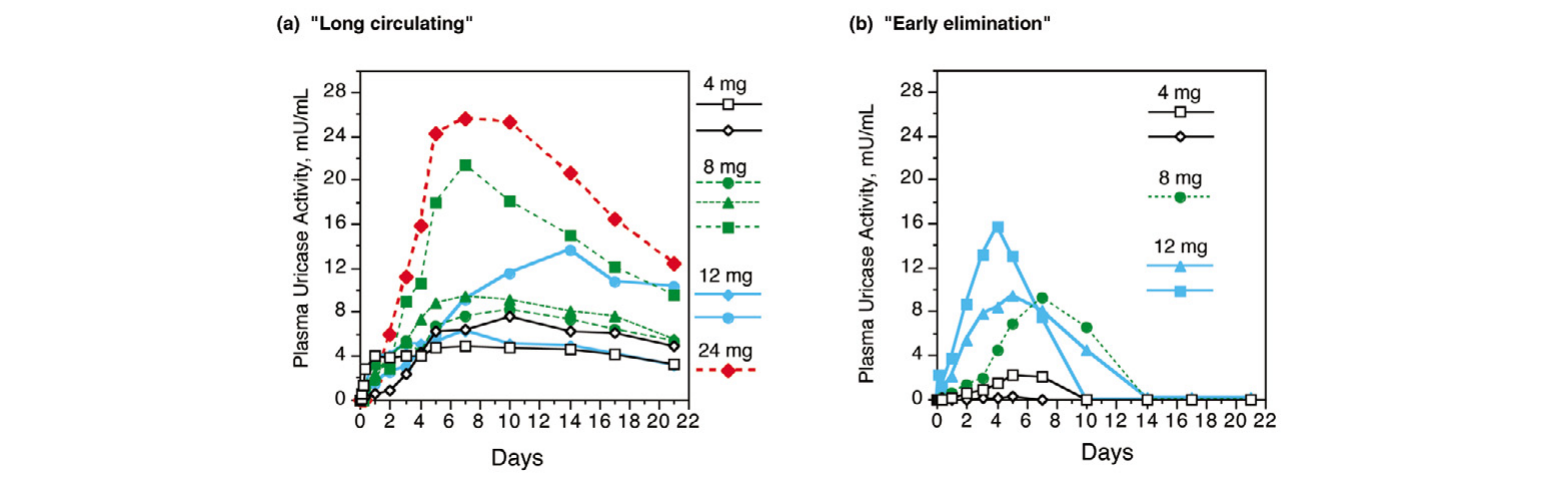
Two pharmacokinetic patterns after single subcutaneous injections of PEG-uricase. **(a) **'Long-circulating' group: eight subjects with uricase activity present in plasma at 21 days after injection. **(b) **'Early elimination' group: five subjects with undetectable plasma uricase activity beyond 10 days after injection. The keys indicate the dose of PEG-modified recombinant mammalian urate oxidase (PEG-uricase).

The time at which uricase activity disappeared from plasma (and area-under-curve calculations, not shown), defined two distinct patterns, which were independent of PEG-uricase dose (Figure 3). In eight subjects ('long-circulating', Figure 3a), pUox was still measurable at three weeks after injection, whereas in five subjects ('early elimination', Figure 3b) pUox could not be detected beyond day 10 after injection. The estimated terminal half-life (*t*_1/2_) of PEG-uricase for the 'long-circulating' group ranged from 10.5 to 19.9 days (*t*_1/2 _could not be accurately determined for 'early elimination' subjects).

Not surprisingly, the effect of PEG-uricase on pUAc was more prolonged in the 'long-circulating' than in 'early elimination' subjects, even though the pre-dose pUAc was higher in the former than the latter (12.3 ± 2.0 versus 9.9 ± 1.6 mg/dl). Mean pUAc declined to about 3.5 mg/dl at day 7 after injection in both groups, but whereas pUAc remained below 6 mg/dl (5.2 ± 3.9) on day 21 in the 'long-circulating' group, pUAc rebounded to more than 7 mg/dl by day 14, and to pretreatment levels by day 21, in the 'early elimination' group (Figure 2b).

### Immunogenicity

The rapid disappearance of pUox in five subjects, some of whom had apparent hypersensitivity reactions (see below), suggested an immune-mediated response to PEG-uricase. An initial screening ELISA performed on 1:100 dilutions of day 0, 14, and 21 sera failed to detect IgG antibodies against unmodified recombinant uricase in any of the 13 subjects (data not shown). A second screening was therefore performed at 1:20 and 1:60 dilutions of plasma, using PEG-uricase as the immobilized antigen. None of the eight subjects in the 'long-circulating' group gave a positive response (data not shown). By contrast, all five 'early elimination' subjects were consistently positive in this ELISA screen, and in other tests for antibody against PEG-uricase.

**Figure 4:**
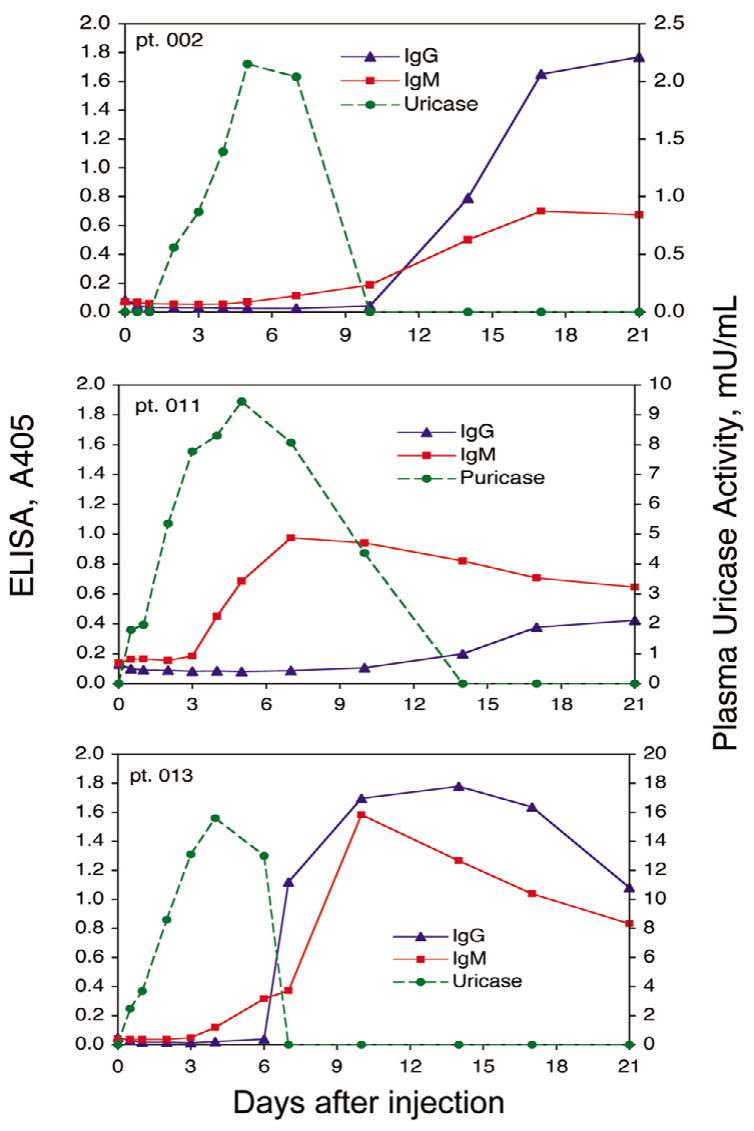
Time course of appearance of IgM and IgG antibodies against PEG-uricase, and of plasma uricase activity, in 'early elimination' subjects 002, 011, and 013. Subject 002 received 4 mg, and subjects 011 and 013 each received 12 mg, of PEG-modified recombinant mammalian urate oxidase (PEG-uricase).

**Figure 5:**
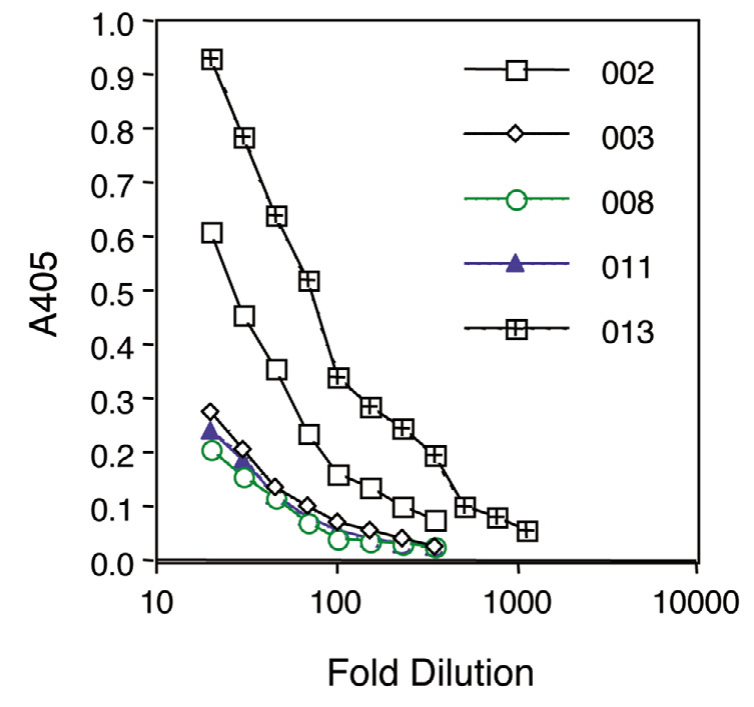
Titer of ELISA for IgG antibody against PEG-uricase. The data shown are for the five subjects who showed early elimination of plasma uricase activity. PEG-uricase, PEG-modified recombinant mammalian urate oxidase.

The evolution of IgM and IgG antibody against PEG-uricase and the relationship to circulating levels of PEG-uricase (pUox) was examined for each subject in the 'early elimination' group. Figure 4 shows results for subjects 002, 013, and 011. In each case, IgM antibody became detectable between 3 and 7 days after injection, preceding the appearance of IgG antibody at between days 7 and 14. End-point titers for IgG antibody against PEG-uricase in day 21 samples of the five 'early elimination' subjects ranged from about 1:110 to 1:310 (Figure 5). The highest titers were found in subjects 002 and 013 in the 4 mg and 12 mg dose cohorts, respectively.

Because IgG antibody was not detected until pUox was declining or undetectable, it might have evolved earlier but have been sequestered in complexes with circulating antigen that were rapidly cleared, or circulating antigen might have prevented antibody from binding to immobilized PEG-uricase in the ELISA. To test the latter possibility we examined the ability of exogenous PEG-uricase to inhibit the ELISA response of day 21 plasma samples from subjects 002, 003, 011, and 013 (in which uricase activity was undetectable). At the highest concentration of PEG-uricase tested in this experiment, which was more than tenfold higher than levels of pUox encountered in the clinical trial, the ELISA signal was still 43 to 77% of that observed in the absence of added PEG-uricase. Therefore, it seems unlikely that circulating PEG-uricase masked the development of anti-PEG-uricase IgG.

### Specificity of anti-PEG-uricase antibody

**Figure 6:**
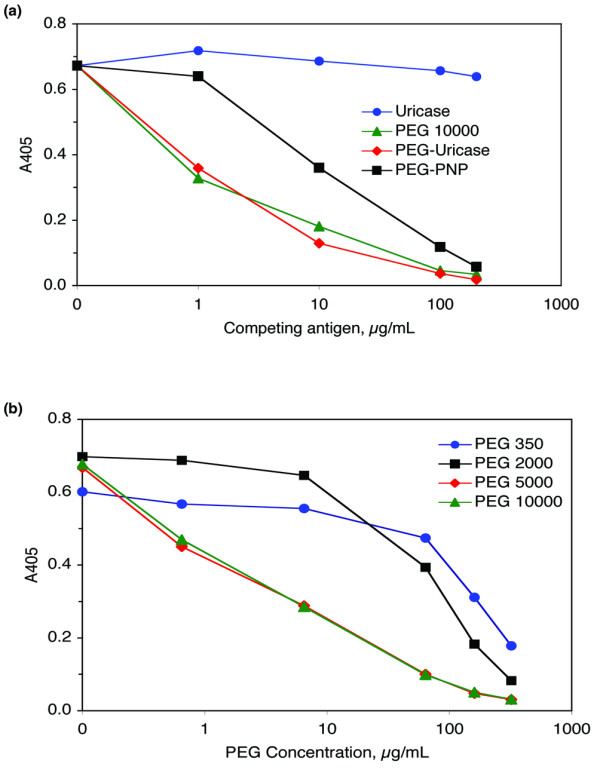
Competition ELISA to determine the specificity of IgG antibody against PEG-uricase. Increasing amounts of the indicated materials were added to aliquots of plasma obtained from subject 013 on day 14 after subcutaneous injection of 12 mg of PEG-modified recombinant mammalian urate oxidase (PEG-uricase). After incubation overnight, these mixtures were tested at a 1:60 dilution in the ELISA for IgG antibody against PEG-uricase. **(a) **'Uricase' is the unmodified recombinant protein used in PEG-uricase, and 'PEG 10000' is 10 K monomethoxyPEG (mPEG) conjugated with glycine instead of uricase protein. 'PEG-PNP' is bacterial purine nucleoside phosphorylase conjugated with mPEG of molecular mass 5 kDa, prepared as described [18]. **(b) **'PEG 350' and 'PEG 2000' are unconjugated, nonactivated mPEGs of molecular masses 350 and 2,000 Da. 'PEG 5000' and 'PEG 10000' are *p*-nitrophenyl carbonate-activated mPEGs of the indicated molecular masses conjugated with glycine.

In the competition experiment shown in Figure 6a, preincubating antibody-positive day 14 plasma from subject 013 with up to 200 μg/ml unnmodified recombinant uricase did not inhibit the ELISA, indicating that the protein moiety of PEG-uricase did not react with anti-PEG-uricase antibody. By contrast, PEG-uricase itself completely inhibited the ELISA, as did 10 K mPEG-glycine, the PEG moiety of PEG-uricase conjugated with glycine instead of enzyme. (In a similar experiment not shown, free 10 kDa PEG diol was as effective an inhibitor of the ELISA as 10 K mPEG-glycine.) Strong inhibition also occurred with PEGylated *Escherichia coli *purine nucleoside phosphorylase, a hexameric bacterial enzyme modified with multiple strands of 5 K mPEG [18].

Lower-molecular-mass PEGs also inhibited the anti-PEG-uricase ELISA, but were less potent than 10 K mPEG (Figure 6b). The approximate concentration necessary to achieve 50% inhibition was 7 μg/ml for 10 K and 5 K mPEG-glycines (0.7 μM and 1.4 μM, respectively), 60 μg/ml (30 μM) for 2 K mPEG, and 160 μg/ml (450 μM) for mPEG of molecular mass 350 kDa (the latter two mPEGs were neither activated nor conjugated with either a protein or an amino acid).

To demonstrate specificity more directly, we developed ELISAs with PEGs, rather than PEG-uricase, to coat the plate. In preliminary experiments, specific signals could be obtained with NPC-activated mPEG and mPEG-glycine, but not with mPEG itself. Because mPEGs, when in solution, did inhibit in competition experiments (for example Figure 6b), unconjugated or non-activated PEG might not bind well to the ELISA plate.

**Figure 7:**
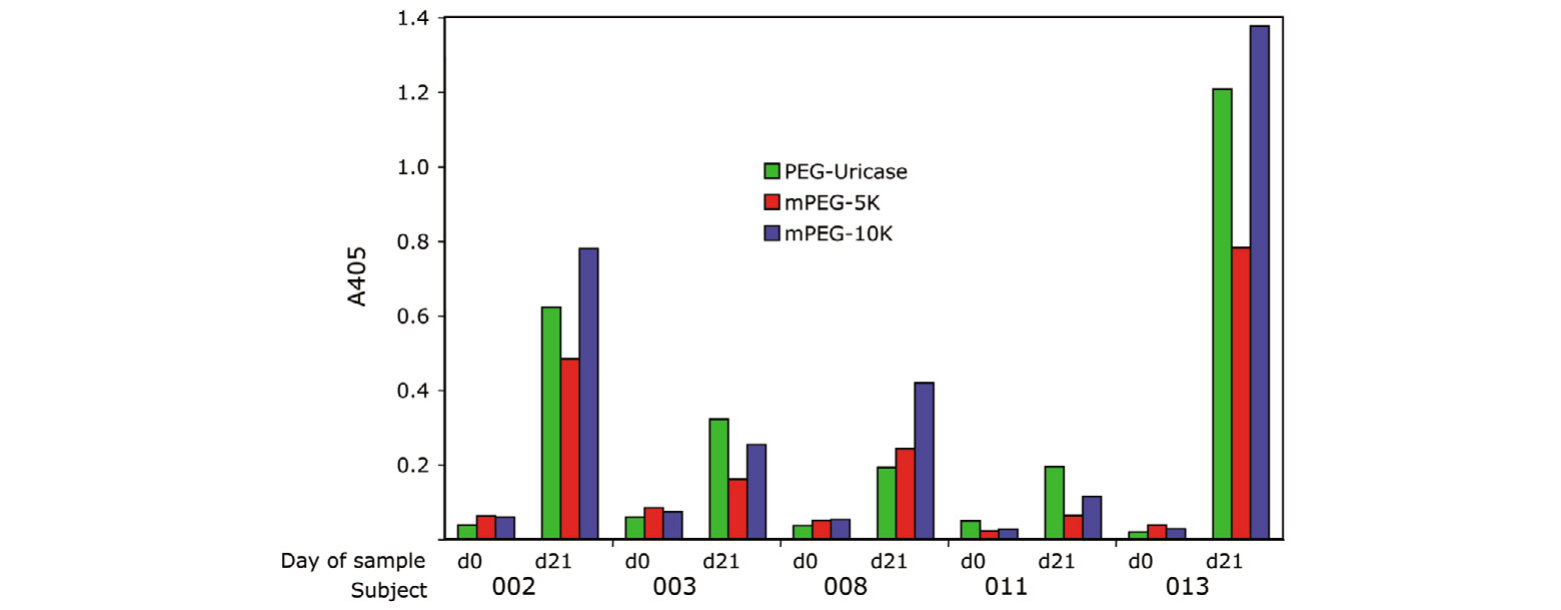
Comparison of ELISAs performed with mPEGs and PEG-uricase. ELISA plates were coated with 2.5 μg of PEG-modified recombinant mammalian urate oxidase (PEG-uricase) or with 25 μg of *p*-nitrophenyl carbonate-activated monomethoxyPEGs (mPEGs) of molecular mass 5 kDa (mPEG-5K) or 10 kDa (mPEG-10K). After washing, the plates were blocked with 1% BSA, 1% glycine in PBS. The indicated plasma samples obtained on day 0 or day 21 after subcutaneous injection of PEG-uricase were then assayed with each substrate at a dilution of 1:50 as described in the Materials and methods section for the PEG-uricase ELISA.

Figure 7 is an experiment in which NPC-activated 5 K and 10 K mPEGs, or PEG-uricase, were used to coat wells of an ELISA plate, which was then used to test for binding of IgG present in the day 0 and day 21 plasma samples from the five 'early elimination' subjects. None of their day 0 samples reacted with any of the three antigens, whereas all day 21 samples reacted with all three antigens, with responses to 10 K mPEG ranging from 0.6 to 2.2-fold those to PEG-uricase. The results confirm that all five subjects had developed IgG antibody against PEG after treatment with PEG-uricase.

### Lack of inhibition of uricase activity by antibody against PEG-uricase

Aliquots of plasma from a subject in the 'long-circulating' group, in which there was uricase activity but had no detectable antibody against PEG-uricase, were mixed with either pretreatment plasma (control) or anti-PEG-uricase-positive plasma that had no uricase activity. The mixtures were then assayed for uricase activity. The expected level of uricase activity was observed, indicating that antibody against PEG-uricase had no inhibitory (neutralizing) effect on PEG-uricase (data not shown).

### Safety and tolerability

Six subjects experienced induration and mild to moderate pain at the injection site within a few hours of subcutaneous injection of PEG-uricase, which resolved within 24 to 48 hours. In addition, three of the five 'early elimination' subjects (one in the 4 mg cohort and two in the 12 mg cohort) developed a second 'late' injection site reaction beginning at 8 to 9 days after injection. In the first case, local swelling and erythema was diagnosed as cellulitis; an antibiotic was administered and the reaction resolved within 48 hours. In the two subsequent instances of late reactions, urticaria appeared at the injection site, and then became widespread within 1 to 2 days. The generalized urticarial eruption was associated with diffuse arthralgia without inflammatory arthritis. No angioedema, respiratory distress, or change in hemodynamic status was observed. The urticaria subsided spontaneously within a few days in one case, and in the other subject after a course of oral prednisone. Six subjects developed gout flares during the 21-day period of observation after injection of PEG-uricase.

## Discussion

### Efficacy of PEG-uricase

In this first phase I trial, single subcutaneous injections of 4 to 24 mg of PEGylated mammalian uricase were administered to 13 markedly hyperuricemic subjects with severe gout. PEG-uricase was slowly absorbed, but by day 7 after injection pUAc had declined by an average of about 8 mg/dl, and had normalized in 11 subjects whose mean pUAc on day 7 had declined to 2.8 ± 2.2 mg/dl. At doses of 8, 12, and 24 mg the mean pUAc at 21 days after injection remained 6 mg/dl or less, which is considered the therapeutic target for management of gout with the currently available drugs to which these patients were refractory or intolerant [19].

The duration of the effect on pUAc correlated with the level and persistence of uricase activity in plasma. Circulating life was independent of dose, and was determined less by the extent of absorption than by the rate of enzyme clearance from plasma. In eight subjects pUox remained detectable for the full 21-day period of observation. The terminal half-life of PEG-uricase in these subjects ranged from 10 to 20 days, or about 13 to 26-fold longer than that reported for the non-PEGylated fungal uricase used to treat acute uric acid nephropathy. These pharmacokinetic and pharmacodynamic findings suggest that PEG-uricase could be very effective for controlling hyperuricemia in subjects with severe, refractory gout.

### Induction of anti-PEG antibodies

IgG antibody against PEG-uricase developed in five subjects at about seven days after injection. Remarkably, these antibodies showed specificity for the PEG rather than the protein moiety of PEG-uricase. The relatively low-titer antibodies did not inhibit uricase catalytic activity but caused a rapid clearance of circulating uricase activity, presumably by crosslinking PEG strands tethered to the enzyme. We speculate that binding of antibody against still unabsorbed PEG-uricase initiated the late injection site reactions observed at 8 to 9 days after dosing in three of these subjects.

The earlier appearance of IgM than IgG antibody (class switching), as well as an apparent anamnestic response observed recently upon re-challenge of an antibody-positive phase I trial subject with PEG-uricase (not shown), are characteristics of a T cell-dependent immune response. Factors that might have promoted this response are the following:

1. Subcutaneous administration and slow absorption would expose PEG-uricase to dermal dendritic cells, which are important in T cell priming.

2. The large size of PEG-uricase (molecular mass about 500 kDa) might have stimulated its pinocytosis or phagocytosis and subsequent processing by dendritic cells, or by macrophages [20,21]. It is unclear at present how flexible and inert PEG molecules might undergo processing.

3. Uric acid crystals have been shown to activate dendritic cells and to act as an adjuvant in mice [22]. This effect in mice, which express uricase, might have been due to hydrogen peroxide, a byproduct of urate oxidation [23]. Either directly or through the generation of H_2_O_2_, extensive deposits of urate crystals in tissues of humans with inadequately treated gout might act as a potent adjuvant to promote an immune response to subcutaneously injected PEG-uricase. H_2_O_2 _derived from urate oxidation might also have caused the transient local inflammation observed in several subjects a few hours after injection.

Erythrocytes have very high levels of catalase, which serves to eliminate H_2_O_2 _generated intravascularly [24]. This potentially protective function, as well as an expectation of improved bioavailability, prompted a second phase I trial of intravenous PEG-uricase. Although confirming the induction of anti-PEG antibodies, no infusion reactions or allergic phenomena were observed in the 24 subjects in that trial (data not shown). Further clinical investigation of intravenous PEG-uricase is in progress.

### Relationship to other PEGylated therapeutics

The clinical value of PEGylation was first shown with PEG-adenosine deaminase (PEG-ADA, Adagen^®^; Enzon Pharmaceuticals) [25], which has been used since 1990 as replacement therapy for immune deficiency due to inherited ADA deficiency. PEGylation has since been used to enhance the therapeutic utility of several other proteins, as well as liposomes, low-molecular-mass drugs, oligonucleotides, lipids, and polysaccharides [11]. Among preparations now in clinical use are PEG-asparaginase for treating leukemia, PEGylated interferons for hepatitis C, PEGylated granulocyte colony-stimulating factor for neutropenia, a PEGylated liposomal doxyrubicin for chemotherapy, and a PEGylated antisense oligonucleotide for macular degeneration.

That PEGylation prolongs the circulating life of proteins is well documented, but it usually does not completely abolish their immunogenicity. Thus, 8 to 9% of PEG-ADA-treated patients develop neutralizing IgG antibodies against ADA protein [26,27]. Antibodies against L-asparaginase, and against interferons α-2a and α-2b, have been observed in a relatively small percentage of patients treated with their PEGylated derivatives [28,29] (and package insert information provided for these products). In the present case, PEGylation does seem to have served the intended purpose of preventing the development of antibodies against uricase, which is a foreign protein in humans.

Antibodies against PEG have been generated in rabbits and mice immunized with PEGylated proteins, including uricase from *Candida utilis*, in the presence of Freund's adjuvant [30-32]. However, in contrast to anti-protein antibodies, a recent review found no reports of PEG-specific antibody in connection with the clinical use of any PEGylated therapeutic in humans [11]. In agreement with this, we have not detected antibodies against PEG in any patient receiving PEG-ADA, including in those treated for longer than a decade (MSH, unpublished data). The immune response to PEG-uricase might conceivably be related to the linkage between mPEG strands and lysine residues of uricase. However, our findings do not suggest such specificity. Thus, we observed reactivity with PEG-PNP, which employs a succinyl linker, with mPEGs linked to glycine by means of a carbamate bond, and with PEGs unlinked to any amino acid.

It is unclear whether systematic testing for anti-PEG antibody has been performed with other PEGylated therapeutics. However, low-titer IgM anti-PEG antibodies were detected in 50% of allergy patients after a 1-year course of allergen immunotherapy with PEGylated ragweed and bee venom allergens; this frequency declined by about half after two years of treatment [33]. It was concluded that anti-PEG antibodies were of no clinical significance. It is interesting that naturally occurring anti-PEG antibodies were detected in about 0.2% of healthy blood donors and 3.3% of untreated allergic patients [33]. From our limited experience with PEG-uricase, it is possible that 'naturally occurring' antibodies that cross-react with PEG could affect the clinical efficacy of some PEGylated proteins.

## Conclusion

In this phase I trial PEGylated mammalian uricase had a prolonged half-life in plasma, and single subcutaneous injections of 4 to 12 mg corrected marked hyperuricemia for up to three weeks in subjects with severe, refractory gout. We observed for the first time in a clinical trial of a PEGylated protein the induction of IgG antibodies against PEG, a phenomenon with possible relevance to other PEGylated therapeutics. Antibodies against PEG-uricase may limit its use in a subset of patients. However, because antibody titers were relatively low, adjustment of dose, route, or schedule of administration may preserve efficacy and limit adverse reactions. With continued treatment, antibody against PEG-uricase may resolve spontaneously in some cases, because animal studies suggest that PEGylated proteins are toleragenic [34-36]. Strategies might also be devised to minimize the immune response. PEG-uricase could provide a more effective method of treating refractory gout than is currently available, and its uricolytic action may provide a more rapid means of resolving tophi than can be achieved by blocking urate synthesis.

## Abbreviations

ADA = adenosine deaminase; ELISA = enzyme-linked immunosorbent assay; HPLC = high-performance liquid chromatography; mPEG = monomethoxyPEG; NPC = *p*-nitrophenyl carbonate; PBS = phosphate-buffered saline; PEG = poly(ethylene glycol); PEG-uricase = PEG-modified recombinant mammalian urate oxidase; pUAc = plasma uric acid concentration; pUox = plasma uricase activity.

ADA = adenosine deaminase; ELISA = enzyme-linked immunosorbent assay; HPLC = high-performance liquid chromatography; mPEG = monomethoxyPEG; NPC = *p*-nitrophenyl carbonate; PBS = phosphate-buffered saline; PEG = poly(ethylene glycol); PEG-uricase = PEG-modified recombinant mammalian urate oxidase; pUAc = plasma uric acid concentration; pUox = plasma uricase activity.

## Competing interests

MSH and SJK, along with scientists from Mountain View Pharmaceuticals, Inc. (Menlo Park, CA), are co-inventors of mammalian PEG-uricase and are among the holders of patents on PEG-uricase. Duke University and Mountain View Pharmaceuticals, Inc., have jointly licensed PEG-uricase to Savient Pharmaceuticals; they could benefit financially if PEG-uricase is approved and marketed. Because of his involvement in the development and licensing of PEG-uricase, MSH did not participate in the recruitment, consenting, or clinical evaluation of trial subjects. MSH's laboratory performed the biochemical and immunologic analyses of coded (anonymous) samples in the course of this clinical trial, and the data generated are reported in this manuscript. JSS, who was not involved in the preclinical development or licensing of PEG-uricase, was the Principal Investigator of this phase I trial. These arrangements were in accordance with restrictions established by the Duke University Medical Center Conflict of Interest Committee.

## Authors' contributions

NJG, SJK, and MSH developed and validated the biochemical and immunologic methods used in this study; NJG and SJK performed these assays and participated with MSH and JSS in analyzing the data reported. ES was the clinical coordinator, and JSS was the Principal Investigator, of the clinical trial. JSS and MSH participated (with personnel from Savient Pharmaceuticals) in designing the clinical protocol. MSH initiated research to develop a PEGylated mammalian uricase for treating refractory gout, directed the laboratory investigations reported, and drafted this manuscript. All authors contributed to the review of the manuscript and have given approval to the final version submitted for publication.

## Figures and Tables

**Table 1:** Characteristics of subjects

Characteristic	Value (*n *= 13)
Sex (F/M)	3/10
Age (years)	
Overall	56.1 (40–75)
Females	64.7 (63–66)
Males	53.5 (40–75)
No. of subjects with tophi	11
No. of subjects on medication	
Allopurinol	6
Uricosurics	0
Colchicine	3
Prednisone (for gout)	8
Serum uric acid (mg/dl)	
Overall	10.1 ± 2.3 (6.9–14.7)
On allopurinol	10.0 ± 2.4 (6.9–14.7)
Not on allopurinol	10.3 ± 2.3 (7.1–13.8)
Serum creatinine (mg/dl)	1.6 ± 0.5 (0.9–2.5)
No. of subjects with co-morbidity	
Osteoarthritis	3
Hypertension	7
Heart disease	2
Diabetes	1

Where errors are shown, results are means ± SD; numbers in parentheses are ranges. Serum uric acid and serum creatinine were measured before allopurinol washout.
